# Calmodulin Mutations Associated with Heart Arrhythmia: A Status Report

**DOI:** 10.3390/ijms21041418

**Published:** 2020-02-19

**Authors:** Walter J. Chazin, Christopher N. Johnson

**Affiliations:** 1 Departments of Biochemistry, Chemistry, and Center for Structural Biology, Vanderbilt University, Nashville, TN 37240, USA; 2 Dorothy M. Davis Heart and Lung Research Institute, The Ohio State Wexner Medical Center, Columbus, OH 43210, USA; 3 Vanderbilt Center for Arrhythmia Research and Therapeutics, Nashville, TN 37232, USA

**Keywords:** calmodulin, Ca^2+^ sensing, Ca^2+^ signaling, ion channels, ion channel regulation, disease associated mutations, LTCC, RyR2, LQT, CPVT

## Abstract

Calmodulin (CaM) is a ubiquitous intracellular Ca^2+^ sensing protein that modifies gating of numerous ion channels. CaM has an extraordinarily high level of evolutionary conservation, which led to the fundamental assumption that mutation would be lethal. However, in 2012, complete exome sequencing of infants suffering from recurrent cardiac arrest revealed de novo mutations in the three human *CALM* genes. The correlation between mutations and pathophysiology suggests defects in CaM-dependent ion channel functions. Here, we review the current state of the field for all reported CaM mutations associated with cardiac arrhythmias, including knowledge of their biochemical and structural characteristics, and progress towards understanding how these mutations affect cardiac ion channel function.

## 1. Introduction

In excitable cells the concentration of calcium ([Ca^2+^]) is an essential signaling mechanism for healthy cellular and organ function [1]. The ubiquitous Ca^2+^ sensing protein calmodulin (CaM) has a prominent role in decoding Ca^2+^ signals as it transduces changes in [Ca^2+^] into biochemical and biomechanical response by altering protein-protein interactions. Ca^2+^ activation of CaM modifies, activates, and de-activates enzymes and ion channels, as well as many other cellular processes. With notable roles in protein phosphorylation and dephosphorylation, cellular Ca^2+^ metabolism, cyclic nucleotide metabolism, gene expression, cell proliferation, muscle contraction, and proteolysis, CaM modification and regulation has implications for nearly every cell in the body [2]. For a detailed discussion regarding the history of CaM discovery and the extensive number of interacting proteins and posited regulatory functions, we refer the reader to Sharma et al. 2018 [2]. For a focused review on the roles of CaM in a cardiomyocyte, see Sorensen et al. 2013 [3]. Despite being one of the most widely investigated proteins, there are still considerable knowledge gaps that limit our understanding of Ca^2+^-induced modification of CaM, and the proteins whose activity is regulated or modified by it [4].

### 1.1. Identification of Human Disease Associated CaM Mutations

Three independent CALM genes produce the same 148 residue CaM protein in all vertebrates [5]. This, combined with its extremely high degree of conservation with even the simplest forms of life, led to the common belief that the fidelity of CaM is essential for life. In 2012, Nyegaard et al. reported describing two patients suffering from ventricular tachycardia and sudden cardiac death. Surprisingly testing for the typical hereditary genetic drivers (RYR2, CASQ2, and KCNJ2, KCNQ1, KCNH2, KCNE1, KCNE2, and SCN5A) failed to identify mutations that could explain the observed disease. However, further analysis revealed alterations to the amino acid sequence of CaM [6], a well-known modulator of cardiac ion channel function [3]. The two mutated CaM proteins (N53I and N97S) were subjected to biophysical characterization, and a reduction in Ca^2+^ binding affinity was observed for the N97S mutation, which supported a central role for CaM mutations in the pathology of disease.

Since this first report, 25 other investigations have identified and/or characterized 18 distinct missense mutations in the CALM genes from patients suffering from heart problems (Table 1). The majority of cases report a Long QT (LQT) and/or Catecholaminergic Polymorphic Ventricular Tachycardia (CPVT) phenotype, with a small subset of disease being classified as Idiopathic Ventricular Fibrillation (IVF). For *CALM*-LQT patients, QTc intervals were found to be highly prolonged with an average of 594 ± 73 ms, and the majority of *CALM*-LQT patients displayed ECG features similar to LQT3 and Timothy Syndrome. *CALM*-CPVT patients suffer from adrenergically-induced cardiac mis-function (such as, ventricular tachycardia and/or ventricular fibrillation). Moreover, the majority of *CALM-*CPVT patient ECG’s display features similar to Anderson Tawil Syndrome [7]. In total, there have been 35 different nucleotide substitutions identified, which have resulted in 28 unique amino acid changes within 74 individual patients [7]. To date, there are no reports that associate any of the CALM mutations with other types of cardiac disease (dilated cardiomyopathy, hypertrophy, or heart failure). However, we note that an awareness of the potential for a person to have a mutation in a CALM gene is relatively recent, and there are likely more examples waiting to be discovered. Moreover, there is limited data to inform on the long-term effects of alterations in the CaM protein sequence. Notably, a collaborative international effort has recently proposed to form a Calmodulinopathy Registry [7]. For an in-depth review of the genetic and clinical findings see Crotti et al. 2019 [7].

### 1.2. The Structure of CaM and Response to Calcium

**Figure 1:**
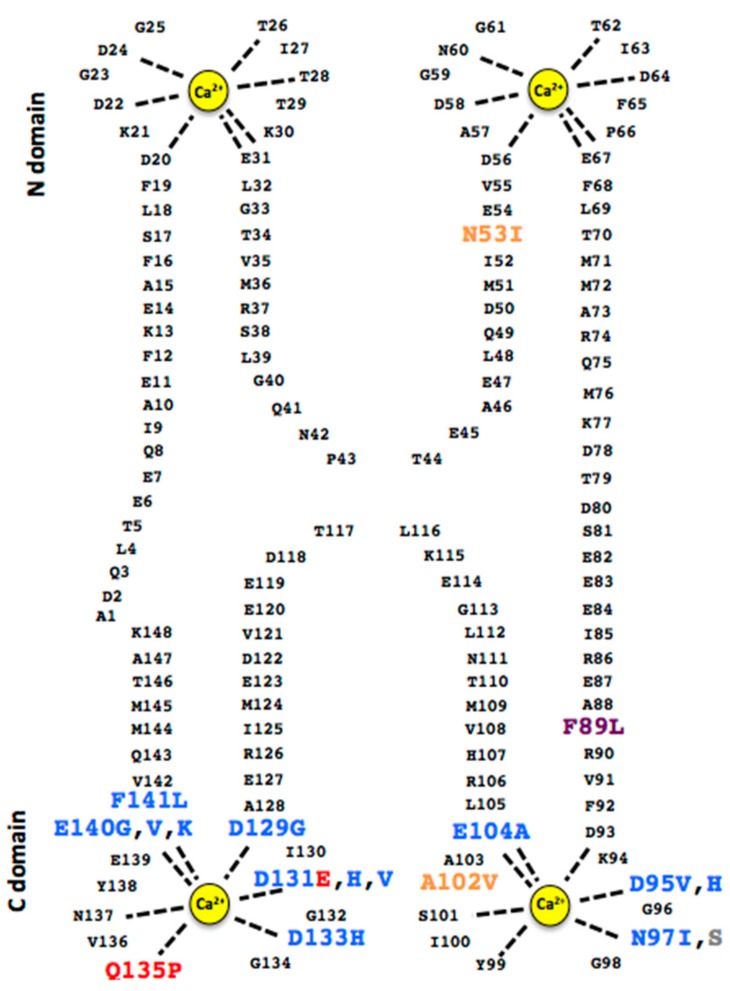
2D schematic of CaM displaying all reported disease associated mutations. Mutations are color coded as follows: **LQTS**, **CPVT**, **IVF**, **bothLQT/CPVT**, **CALM1 = CPVT, CALM2 = LQTS**.

The CaM protein has two globular N- and C-terminal domains (CaM-N, CaM-C) connected by a flexible linker (Figure 1). Each domain is comprised of two EF-hand helix-Ca^2+^ binding loop-helix motifs. Within each domain, the EF hands are oriented in a face–face manner and create a structurally stable 4-helix bundle. The stability of the domains arises from substantial hydrophobic cores formed from residues contributed by both EF-hand motifs (I and II in CaM-N, III and IV in CaM-C), complemented by a β-sheet type interaction between the two Ca^2+^ binding loops. In the absence of Ca^2+^, the domains adopt a compact closed conformation with hydrophobic residues packed internally [27]. CaM with no Ca^2+^ bound will nevertheless interact with IQ motif (IQxxx[R,K]Gxxx[R,K]) sequences in target proteins [28], accompanied by a shift to a “semi-open” conformation. At low resting-state levels of calcium, IQ motif interactions serve to pre-localize CaM to the intended target protein so it is poised in the correct position when Ca^2+^ signals are generated. The binding of Ca^2+^ in the EF-hand loops causes a substantial reorientation of the helices, which results in an opening of the domain and exposure of a significant hydrophobic surface [27,29]. This hydrophobic surface exposure is the key to the on-off triggering of interactions between CaM and its many cellular targets [30]. Cooperativity in the binding of Ca^2+^ within each domain and differences in Ca^2+^ k_on_/k_off_ rates between the domains make CaM an effective Ca^2+^ sensing protein over a range of concentrations (μM to mM) [31,32,33,34].

### 1.3. Overview of Cardiomyocyte Ca^2+^ Homeostasis

At the start of a cardiac action potential, intracellular [Ca^2+^] is low (~0.1 μM). In response to membrane depolarization (initiated by voltage-gated sodium channels); voltage-gated Ca^2+^ channels allow passage of Ca^2+^ ions into the cytosol in the near vicinity of RyR2. This activates a Ca^2+^ induced Ca^2+^ release mechanism in which the cardiac ryanodine receptor (RyR2) releases Ca^2+^ from the sarcoplasmic reticulum (SR) in quantities sufficient to elevate bulk cytosolic [Ca^2+^] from ~0.1 μM to ~1 μM. In cardiomyocytes, elevation of [Ca^2+^] is sensed by troponin C and this initiates muscle contraction via the sarcomeric proteins. Bulk cytosolic [Ca^2+^] is then reduced to lower levels (~0.1 μM) by the SR Ca^2+^-ATPase (SERCA), which pumps Ca^2+^ back into the SR where it is bound to the Ca^2+^ buffering protein calsquestrin [35].

While this overall scheme of cardiac Ca^2+^ homeostasis is widely accepted, the timing in which each protein encounters a [Ca^2+^] transient likely varies based on protein location within a cardiomyocyte. Cellular architecture and micro-domain structure are believed to influence and/or tune changes in [Ca^2+^] and this topic is the subject of much active investigation [36]. Understanding these relationships is paramount for understanding the timing in which CaM will modify and influence the function of a protein.

### 1.4. An Overwhelming Number of CaM Interactions

CaM has been documented to interact with hundreds of proteins, which in turn modifies a wide range of cellular processes [2,30,37]. As expected, with so many CaM interactions and functions, any alterations in CaM has potential implications for nearly every cell in the body. Given that thus far the phenotypes observed in patients containing mutations in the CALM genes are predominantly resulting from electrical cardiac disorders, we focus here on CaM and key CaM-modified cardiac ion channels, the L-type Ca^2+^ channel (Ca_V_1.2) and the cardiac ryanodine receptor (RyR2). We also include some consideration of CaM-mediated post-translational modifications by CaM Dependent Kinase (CaMKII) and CaM stimulated phosphatase Calcineurin (CaN). Even within this highly focused context, it is clear that understanding the full range of consequences of CaM mutation within normal physiology as well as disease will require far more research than can be afforded by a single investigator. As such, we acknowledge and highlight a fundamental need for global collaboration and communication to accomplish such foundational work.

## 2. Reported Molecular Effects of CaM Mutations

It is well established that LQT and CPVT can be caused by improper function of Ca_V_1.2 and RyR2 [38]. Early investigations quickly demonstrated that CaM mutations altered interactions and gating properties of these ion channels [6,8]. However, thus far, attempts at segregating and correlating phenotype with gene modification or the protein mutation identity, location or Ca^2+^ binding affinity have been met with only modest success [8]. In hindsight, this is not overly surprising given the many activities of CaM, and the substantial knowledge gaps surrounding the mechanisms by which CaM modifies the gating of different ion channels (*vide infra*).

### 2.1. Alterations in CaM Ca^2+^ Affinity

Since CaM is such a well-known Ca^2+^ sensor, most studies of disease associated mutations include a measure of the Ca^2+^ affinity (Table 1). Most groups use a method that monitors the intrinsic fluorescence of Tyr and Phe residues over the course of a Ca^2+^ titration [14,31,39]. In this approach, the data are fit to the Adair equation, with the increase in fluorescence of Tyr attributed to Ca^2+^ binding in the CaM-C domain, and the decrease in fluorescence of Phe attributed to Ca^2+^ binding in CaM-N (see Ferrell et al. for assumptions regarding co-cooperativity and use of the Adair equitation [40]). This approach is widely adopted because it does not require modification of CaM or use of probes that could bias the measurements.

It is important to note that there are limitations with respect to the interpretation of these data and the ability to correlate the results to in vivo functional data. At the outset, the measurement of Ca^2+^ affinity for isolated CaM protein is inherently limited because it is well known that binding of Ca^2+^ is energetically coupled to the binding to a target protein. Hence, the Ca^2+^ affinity measured for the isolated CaM can be substantially different from the affinity in the functional context [41]. This increases the uncertainty in predictions of ion channel dysfunction based on the Ca^2+^ affinity measured for the isolated mutant CaM. At a technical level, some caution is also needed for interpretation of the Ca^2+^ affinities measured for the CaM-N domain. In wild type CaM, changes in Phe fluorescence can be correlated with Ca^2+^ binding in CaM-N because the Phe residues in CaM-C are structurally arranged such that their overall fluorescence is quenched and they do not contribute to the observed signal [31,39]. Therefore, if a mutation perturbs the arrangement of the Phe residues in CaM-C, it may no longer be possible to attribute all of the observed Ca^2+^-induced changes in Phe fluorescence to binding in CaM-N. The need for concern is underscored by NMR analysis of CaM mutants, as the spectra of a number of the individual disease associated mutations revealed significant perturbations relative to the wild type protein [14,24]. These data suggest the possibility that the mutations may modify the local environment of an extensive number of residues in CaM-C. One additional technical concern is that great care must be taken in normalizing Ca^2+^ titration data, as this process can mask incomplete saturation of the four Ca^2+^ binding sites in CaM [22] and therefore compromise quantitative comparison of Ca^2+^ affinities.

### 2.2. CaM Binding to Target Proteins

The canonical structure of CaM bound to a peptide or portion of a target protein has the two domains wrapped around the target [30]. Over the years, the dynamic nature of CaM interaction has been increasingly recognized, in part due to solution based experiments demonstrating that CaM can engage protein targets using a myriad of different binding configurations with its two domains [42,43]. For example, we have recently shown that CaM can utilize an elongated configuration where its two domains simultaneously engage two distinct target sites contained within a cytosolic loop of a voltage gated sodium channel [44]. Others have speculated that similar elongated configurations are likely also utilized by CaM for modulation of other ion channels such as the Ca_V_ channels [45]. CaM is notoriously promiscuous and there are an abundance of reports describing interactions with hydrophobic peptides or fragments of proteins. Until recently, experimental limitations have restricted structural characterization of CaM interactions to fragments of target proteins with a few exceptions. In this vein, recent advances in CryoEM are enabling a leap forward in structural analyses by greatly facilitating characterization of CaM bound to intact ion channels [46,47].

Studies of the affinity of CaM for Ca^2+^ or for its targets has been the primary focus of biophysical investigations, but due to the coupling of these binding events, this perspective is insufficient to completely understand the system. Because each CaM domain can engage a unique binding site and the two domains are linked together by a flexible tether [48], high overall affinity can be attained while maintaining the ability for rapid response (high rates of effective k_on_, and k_off_). The pre-localization of CaM through binding to IQ motifs also enhances the effective k_on_. These factors contribute significantly to the rapid CaM association and dissociation from a protein target required for cardiac ion channel function. In the cellular context, faster k_off_ rates at each CaM domain-binding interface should allow for enhanced sampling of cytosolic conditions, *i.e*., more frequent Ca^2+^ sensing. Given the intricacies of the kinetics, as well as the aforementioned target protein influencing the affinity of CaM for Ca^2+^, predicting how a CaM mutation modulates ion channel function and cardiac physiology is not yet possible. The challenge is best exemplified in a study that showed even with atomic resolution structures, one is still unable to predict a dose response for Ca^2+^ binding by CaM [49]. Nevertheless, substantial insights into the origin of the dysfunction of disease associated CaM mutations can be obtained through detailed experimental analyses.

### 2.3. CaM Modification of Ca_V_

CaM has a prominent role in inactivation of cardiac Ca_V_ conduction termed CaM Dependent Inactivation (CDI) [45]. CDI has been the subject of immense investigation and functionally, the phenomenon is well characterized in cells and animal models. Conversely, the structural details of CaM interaction with full-length Ca_V_’s are only partially illuminated [50], and the mechanism describing how CaM binding alters channel structure and inactivates ion conduction requires further investigation [45]. Interrogation of CDI for several of the CaM mutations demonstrated loss of CDI as a causative mechanism of the observed LQT [9]. Mutations in just one of the CaM genes can produce a dominant negative effect (exert an influence in the presence of endogenous wild type CaM) because these mutants bind to Ca_V_ with similar affinity as wild type CaM in the absence of Ca^2+^, which suggests they pre-localize to the channel as well as the wild type protein [9].

### 2.4. CaM Modification of RyR2

CaM modification of RyR2 is less well-defined, presumably because the effect is smaller in amplitude relative to Ca_V_ channels, and potentially dependent upon other channel modifiers. Nevertheless, there is a general consensus that CaM interactions with RyR2 reduce Ca^2+^ release. This is typically observed as a reduction in the frequency of Ca^2+^ sparks, i.e., small aliquots of Ca^2+^ that are released during diastole [51]. In times of stress or in certain disease states, RyR2 spark frequency is enhanced [52,53]. Conversely, increased spark frequency is considered by most as a hallmark of disease, as aberrant Ca^2+^ can initiate arrhythmia and/or untimely cardiac contraction [38]. Several of the disease-associated CaM mutations result in an increase in RyR2 spark frequency (Table 1). No clear trends for CaM mutants were apparent in the properties of RyR2 function within cardiomyocytes, which could be used to predict the effect of the individual CaM mutations on RyR2 function. However, in a recent report using a model with RyR2 transfected into HEK293 cells; 14 out of 14 disease associated mutant CaM’s exhibited both diminished CaM inhibition of RyR2 Ca^2+^ release, and increased store overload Ca^2+^ induced release [54].

The interaction of CaM with RyRs have been the subject of intense investigation, particularly with respect to the location on RyRs and the configuration of CaM. However, attempts to relate these data to cardiac function have been controversial. A crystal structure of CaM bound to an RyR1 peptide suggested CaM engages the channel using a wrap-around configuration [55]. Subsequent studies provided evidence for CaM interacting with the intact RyR2 channel in an elongated configuration and raised the possibility of a second CaM binding site [56]. A leap forward in understanding has been made based on recent studies using Cryo-EM of the intact channel: several structures of full length RyR2 in complex with either apo-CaM or Ca^2+^-CaM in the absence and presence of caffeine and other cofactors have been reported [57]. These structures reveal changes in the RyR2 channel pore diameter that are consistent with CaM reducing diastolic RyR2 Ca^2+^ conduction. Moreover, in the absence of Ca^2+^, the backside surface of CaM-N (opposite the hydrophobic pocket typically engaged in target interactions) is used to bind to RyR2. Remarkably, this type of CaM interaction was predicted by Ikura and coworkers more than 20 years ago in their report of the ab. initio solution structure of Ca^2+^-free CaM [27].

The detailed mechanism for how CaM allosterically modifies the channel pore diameter is currently unknown and requires further investigation. In addition, there remain considerable knowledge gaps in understanding how other RyR2 accessory proteins and ligands influence and modify the interaction of CaM with RyR2 and in turn the conformation of the receptor. Intriguingly, CaM modification of RyR2 appears to be dependent on CaMKII phosphorylation of the channel [58]. This adds an additional layer of complexity as several of the disease associated CaM mutations have been shown to reduce CaMKII activity in vitro [11].

### 2.5. Improving Treatment Options for Calmodulinopathies

While ion channels responsible for acute calmodulinopathy disease have been identified, current treatment options are considered disquietingly insufficient [7]. Improving treatment options and long term health care will likely require a more detailed understanding of the mechanisms by which CaM modifies ion channel gating. Currently it is not possible to predict the consequences of a CaM mutation with respect to a specific ion channel function; and even less so the impact to cellular function or physiology. Table 2 lists the current state of understanding of the possible effects of a disease associated CaM mutation on ion channel gating and the likely physiological consequence.

In silico modeling has made limited progress in this arena, and extensive experimental data are necessary for evaluating each CaM-protein system [59]. Overall, despite immense investigation, there is a limited understanding for how individual CaM-ion channel interactions transduce changes in intracellular [Ca^2+^] into modified ion channel gating and in turn how this is integrated into overall cardiac function.

Given the diversity of cellular processes influenced by CaM, loss of, untimely, or improper function of a CaM-modified protein can also elicit consequences beyond cardiomyocyte function. For example, RyR2 calcium handling plays a role in other metabolic pathways. In pancreatic β cells, alterations to the activity of the RyR2 channel and its calcium release have been shown to have implications for insulin release and glucose homeostasis [60]. Exploration of such associations may provide insight into novel mechanisms of disease, such as connections between insulin resistance, aberrant calcium handling and arrhythmia, which have been observed in patients with altered glucose homeostasis and insulin resistance [60]. Clearly, a substantial leap forward in understanding fundamental CaM-mediated processes is required before reliable prediction of the effects of CaM mutation with respect to molecular, cellular, and physiological function can be made.

Excitingly, the emergence of structural data describing more complete CaM-ion channel interactions is affording insight into the mechanisms of CaM channel modification. Moreover, atomic resolution data is allowing for structure-designed mutations that can interrogate each CaM interaction as an isolated variable with respect to ion channel function. In turn, these data provide a foundation that is necessary to interpret highly complex kinetic rate data of Ca^2+^-CaM-protein interactions. All three types of data (structure, function, and kinetic rates) are necessary for understanding CaM modification of a target protein, and how a CaM mutation can compromise the cellular process. Interdisciplinary investigations of this type can elucidate the mechanisms CaM uses to transduces changes in [Ca^2+^] into modified ion channel function. Such studies will aid understanding how changes in CaM protein sequence can be tolerated, cause or contribute to disease, or potentially even someday enhance physiology [61].

## 3. Concluding Remarks

The discovery of mutations in the CaM protein was rather surprising to the many investigators who have been studying CaM structure and function over many decades. As is evident from the large number (now approaching 30) of mutations with clear disease association, the current paradigm has changed. At the most fundamental level, there is an urgent need to better understand the degree of intrinsic variation of gene sequence in all genes and the exact origins of having multiple copies of the same gene in a given organism. In the context of this review, one long-term objective for the field is to understand the extent of sequence variation and how it affects CaM function in cardiac physiology and disease pathology. In the meanwhile, efforts such as the proposed Calmodulinopathy registry are paving the way for research laboratories and physicians to connect to each other on a global scale and foster investigations aimed at improving treatment options for patients suffering from syndromes driven by CaM mutations.

## Figures and Tables

**Table 1:** Reported biophysical effects of disease associated calmodulin (CaM) mutations.

Protein Mutation	Mutated Gene	CaM-C Ca^2+^ Affinity (μM)	Ca_V_ C.D.I.	CaM-RyR Interaction	RyR2 Sparks	RyR2 Waves	RyR2 Open Probability	RyR2 ryanodine Binding ^a^	CaMKII Activity
N53I	*CALM1* [6]	3.1 ± 0.2 [8]	= WT [9]	= WT(6) 🡅 RyR2 binding [10]	🡅 fq [8]	🡅 fq [8]	🡇 O.P. at low and high [Ca^2+^] [8]	🡅 I.E. [10]	= WT CaM [11]
F89L	*CALM1* [12]	17 ± 0.6 ^^^		🡇 RyR2 binding at low and high [Ca^2+^] [13] *				🡇 I.E. [13]	🡇 WT CaM [11]
D95V	*CALM2* [14]	38 ± 6 [14]	loss of C.D.I. [9]	🡅 RyR2 binding [10]		= WT fq [8]	= WT CaM O.P. [8]	🡅 I.E. [10]	🡇 WT CaM [11]
N97I	*CALM2* [15]	15 ± 1 [15]							
N97S	*CALM1* [6] *CALM2* [15,16,17]	11 ± 1 [8]	partial C.D.I.[9]	🡇 at low [Ca^2+^] [6] = WT CaM [10]	🡅 fq [8]	🡅 fq [8]	🡅 O.P. at high [Ca^2+^] [8]	= WT CaM [10]	🡇 WT CaM [11]
A102V	*CALM3* [18]	7.3 ± 1 [18]	partial C.D.I. [18]	= WT CaM [18]	🡅 fq [18]	🡅 fq [18]			
E104A	*CALM1* [19]	29 ± 1 ^^^							
D129G	*CALM1* [14] *CALM2* [20] *CALM3* [21,22]	150 ± 30 [14]	loss of/diminished C.D.I. [9,23]	🡇 RyR2 binding [10]		🡇 fq [8]		= no CaM [10]	can bind CaMKII but does not activate [11]
D129V	*CALM2* [20]								
D131E	*CALM2* [15]	48 ± 10 [15]							
D131H	*CALM2* [24]	177 ± 48 [24]	impaired [24]						
D131V	*CALM1* [24]	146 ± 61 [24]	impaired [24]						
D133H	*CALM2* [15]	27 ± 5 [15]							
Q135P	*CALM2* [15]	19 ± 2 [15]							
E140G	*CALM1* [20]	27 ± 2 [20]	dominant loss of C.D.I. [20]		= wt [20]				
E140K	*CALM3* [22]	75 ± 7 [22]							
E140V	*CALM1* [22]	54 ± 4 [22]							
F141L	*CALM1* [14,20]	15 ± 0.5 [14]	loss of /impaired C.D.I. [9,25]	= WT CaM [10], 🡇 CaM-RyR Ca^2+^ binding [26]		🡇 fq [8]	🡇 O.P. compared to WT CaM [26]	= WT CaM [10]	= or 🡅 WT CaM [11]

Wild Type (W.T.); frequency (fq); Open Probability (O.P.); Inhibitory Effect (I.E.); ^a^ Compared to WT CaM reduction of RyR2 ryanodine binding. * Additional fusion cleavage residues added to CaM can modify CaM interactions, ^ Johnson & Chazin et al. unpublished data. ^#^ WT CaM CaM-C domain Ca^2+^ affinity = 2.5 ± 0.5 μM. Blank spaces indicate data is not available.

**Table 2:** Potential Mechanisms of CaM dysfunction.

Mutation Effect	Molecular Effect	Predicted Health Impact	Rational	Predicted Outcome
Defective apo CaM binding: i.e., loss of apo CaM protein interaction	CaM is not pre-localized and poised for interaction when Ca^2+^ signals.	likely benign	Because CaM is encoded by three genes so there is a strong possibility that other endogenous CaM can serve as a Ca^2+^ sensor	At worst this type of mutation could result in loss of CaM modification of a protein target
Normal apo CaM interaction with defective Ca^2+^ CaM interaction	Apo CaM can engage the target protein, but does not engage protein correctly in presence of Ca^2+^	likely pathogenic	This type of mutation can display a dominant negative effect.	This type of mutation could occupy several CaM targets and impair or inhibit Ca^2+^ modification
Normal apo and Ca^2+^ CaM interactions with defective Ca^2+^ sensing	Both apo and Ca^2+^ CaM binding can be achieved. Rates of Ca^2+^ sensing are adjusted causing a timing dysfunction.	likely pathogenic	Could cause loss of, or delay CaM modification	Depending on the role of CaM, this could result in: loss of channel availability, or untimely channel conduction
Enhanced Apo CaM interaction with defective Ca^2+^ sensing	Mutant CaM can out compete endogenous CaM so a greater population is pre-localized to the target protein	potentially most pathogenic	This would impair the maximal amount of endogenous CaM from binding and has the potential to be most catastrophic	Such mutations are likely to be incompatible with physiology and presumable removed through natural selection

## Abbreviations

[Ca^2+^]	Calcium concentration
CaM	Calmodulin
Ca_V_	Voltage gated calcium channel
CPVT	Catecholaminergic Polymorphic Ventricular Tachycardia
IVF	Idiopathic Ventricular Fibrillation
LQT	Long QT
RyR2	Ryanodine Receptor Type 2
